# Upregulation of DACT2 suppresses proliferation and enhances apoptosis of glioma cell via inactivation of YAP signaling pathway

**DOI:** 10.1038/cddis.2017.385

**Published:** 2017-08-10

**Authors:** Ying Tan, Qiu-Meng Li, Ning Huang, Si Cheng, Guan-Jian Zhao, Hong Chen, Song Chen, Zhao-Hua Tang, Wen-Qian Zhang, Qin Huang, Yuan Cheng

**Affiliations:** 1 Department of Neurosurgery, The Second Affiliated Hospital of Chongqing Medical University, Chongqing, China; 2 Department of Orthopaedics, The Second Affiliated Hospital of Chongqing Medical University, Chongqing, China; 3 Department of Neurosurgery, The First Affiliated Hospital of Chongqing Medical University, Chongqing, China; 4 Department of Gynaecology and Obstetrics, The Second Affiliated Hospital of Chongqing Medical University, Chongqing, China

## Abstract

DACT2, one of the Dact gene family members, was shown to function as a tumor suppressor. However, its function in gliomas remains largely unknown. In this study, we investigated the role of DACT2, underlying molecular mechanisms and its clinical significance in glioma patients. Downexpression of DACT2 in gliomas compared with adjacent normal brain tissues was correlated with glioma grade and poor survival. Cox regression analysis revealed that the DACT2 is an independent prognostic indicator for glioma patients. Overexpression of DACT2 in glioma cells inhibited proliferation, cell cycle and enhanced apoptosis, sensitivity to temozolomide *in vitro* and suppressed tumor growth *in vivo*. Whereas knockdown of DACT2 induce opposite reaction. Mechanistically, overexpression of DACT2 resulted in upregulation of important signaling molecules such as p-YAP and p-*β*-catenin, and prevent YAP translocating into nucleus and sequestering in the cytoplasm to degrade. The study further proved that DACT2 can suppress YAP through Wnt/*β*-catenin signaling pathway. Collectively, these data indicate that DACT2 has a tumor suppressor function via inactivation of YAP pathway, providing a promising target for the treatment of gliomas.

Gliomas, the most aggressive and common primary brain tumor, account for over 32% of all brain tumors and approximately 80% of malignant primary brain tumors.^1, 2, 3, 4^ Current standard of medical treatment for gliomas includes adjuvant chemotherapy and radiotherapy after maximal safe resection, however, median overall survival of glioma patients is not more than 12–18 months after the diagnosis.^5, 6, 7^ It is an urgent need for seeking the exact molecular mechanisms of the glioma progression and developing new and effective therapeutic targets to improve the patient survival.

Dapper homolog 2 (DACT2) belongs to DACT gene family, located on human chromosome 6q27 which is a region frequently related to loss of heterozygosity in human cancers.^8, 9, 10, 11, 12^ There are three members in DACT family, including DACT1, 2 and 3.^13^ DACT1 is located on human chromosome 14q22.3 and has been revealed frequently to be methylated in hepatocelluar carcinoma. DACT3 is located on chromosome 19q13.32 and is reported to be tightly regulated by histone modification in colorectal cancer.^14, 15^ However, compared with DACT1 and 3, the function of DACT2 in tumorigenic signaling and development has not been fully clarified.

In previous studies, DACT2 was found to be methylated in lung cancer and DACT2 methylation enhanced lung cancer proliferation.^16^ In human hepatocelluar carcinoma, loss or reduction of DACT2 expression is correlated with promoter hypermethylation and it promotes cell proliferation, induced cell cycle arrest in cell lines.^17^ Several studies have reported DACT2 methylation activated Wnt/c-Jun signaling and Wnt/*β*-catenin signaling pathway which played a critical role in cell proliferation, apoptosis, invasion and migration.^18, 19, 20, 21, 22^ On the other hand, the biological roles of DACT2 on the gliomas and its clinical significances have not yet been elucidated.

YAP (Yes-associated protein), the key downstream transcription factor of the Hippo signaling pathway which has pivotal roles in the regulation of the cell proliferation, cell cycle, apoptosis, differentiation, survival,^23, 24, 25, 26^ has been reported to be elevated in various human cancers including colorectal carcinoma,^27^ gastric cancer,^28, 29^ liver cancer,^30, 31^ breast cancer,^24^ pancreatic cancer,^32^ lung cancer,^33, 34^ ovarian cancer.^35^ In addition, overexpression of YAP was found in gliomas, and correlated with poor overall survival of glioma patients.^36^ Function as a transcription factor, YAP shuttles between the cytoplasm and the nucleus. When YAP was phosphorylated, it will be sequestered in the cytoplasm and degraded.^37^ Conversely, YAP will translocate into nucleus when it was unphosphorylated and promote transcription of growth promoting or apoptosis inhibiting genes.^38, 39^ Most previous studies mainly focused on the effect of DACT2 on the Wnt/*β*-catenin signaling pathway that plays an important function in development of various tumors. Besides, lots of studies demonstrated that there was the intersection between the Wnt signaling pathway and YAP signaling pathway in contributing progression of tumor.^40, 41, 42, 43^ However, there is no study about whether DACT2 regulates YAP signaling pathway which is also critical for tumor development.

In the present study, we explored the biological impact of DACT2 on the progression of glioma and explored the mechanisms underlying its tumor suppressor role. We showed that DACT2 is downregulated in glioma tissues and correlated with poor survival. Overexpression of DACT2 resulted in upregulation of Bax, p-YAP and p-*β*-catenin, downregulation of PCNA, CyclinD1, YAP and *β*-catenin, and prevent YAP translocating into nucleus and sequestering in the cytoplasm to degrade. The study further proved that DACT2 can suppress YAP through Wnt/*β*-catenin signaling pathway.

## Result

### Downregulation of DACT2 correlates with progression and poor prognosis in gliomas

**Figure 1:**
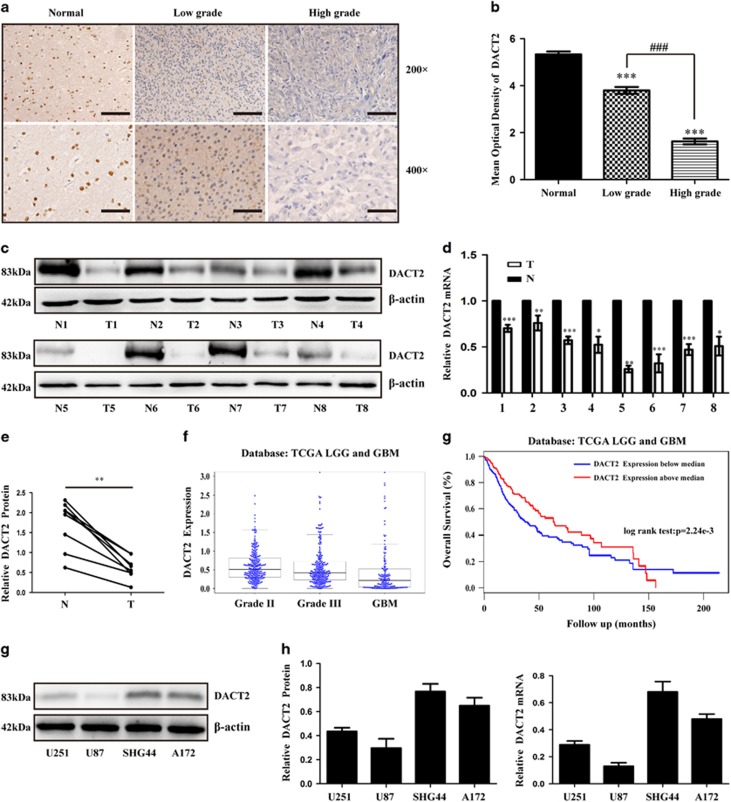
Low DACT2 expression predicts a poor prognosis for patients with glioma. (**a**) Representative images of DACT2 immunostaining in normal brain tissues, low-grade glioma tissues (WHO I-II) and high-grade glioma tissues (WHO III-IV). Low power (200 ×) scale bars: 100 *μ*m, high power (400 ×) scale bars: 50 *μ*m. (**b**) Statistical quantification of the mean optical density in normal brain tissues, low-grade glioma tissues, and high-grade glioma tissues (***P<0.001, ^###^*P*<0.001). (**c**–**e**) Western blot and qRT-PCR analysis shows DACT2 expression was lower in 8 pairs of glioma tissues (T) compared to paired adjacent non-tumor tissues (N) (*P<0.05,** *P*<0.01, ***P<0.001). (**f**) DACT2 expression in glioma was decreased as the grade of glioma incresed from the TCGA LGG and GBM data sets (*N*=699, *P*<0.0001) (**g**) Survival analysis of patients with glioma from the TCGA data sets (*N*=699, *P*<0.01). H., I., J. Western blot and qRT-PCR analysis shows endogenous expression of DACT2 in four glioma cell lines

We first examined the protein levels of DACT2 in 80 gliomas tissues and 10 normal brain tissues by immunohistochemistry (IHC), the result showed all normal brain tissues expressed DACT2 at higher levels, while glioma tissues with different grades showed apparently lower levels of DACT2 expression (Figures 1a and b). Next, we assessed the mRNA and protein expression of DACT2 in eight glioma tissues and paired adjacent tissues. qRT-PCR and Western Bolt revealed that DACT2 was significantly downexpressed in glioma tissues compared with the paired adjacent tissues at mRNA and protein levels (Figures 1c–e).

In addition, The Cancer Genome Atlas (TCGA) glioma data set also showed lower expression of DACT2 in higher grade glioma (Figure 1f). Then, the relationship between DACT2 expression and various clinicopathological features of glioma tissues was analyzed. *χ*2 test revealed that expression levels of DACT2 significantly correlated with the WHO grade, Karnofsky Performance Score (KPS) and age (*P*<0.01; Supplementary Table 1). However, there were no statistical significances in gender. Furthermore, the impact of DACT2 expression on the prognosis in gliomas patients was investigated by the TCGA data sets. The Kaplan–Meier curve revealed that patients with lower DACT2 expression had a significantly poorer overall survival (OS) compared to patients with higher DACT2 expression in gliomas with different grades (Figure 1g). Moreover, Univariate and multivariate analyses were used to determine whether the expression levels of DACT2 and various clinicopathological characteristics were independent prognostic parameters. The result demonstrated that DACT2 expression, WHO grade, KPS and age were independent prognostic parameters for glioma patients (Supplementary Table 2).

### DACT2 overexpression inhibits growth of gliomas *in vitro*

**Figure 2:**
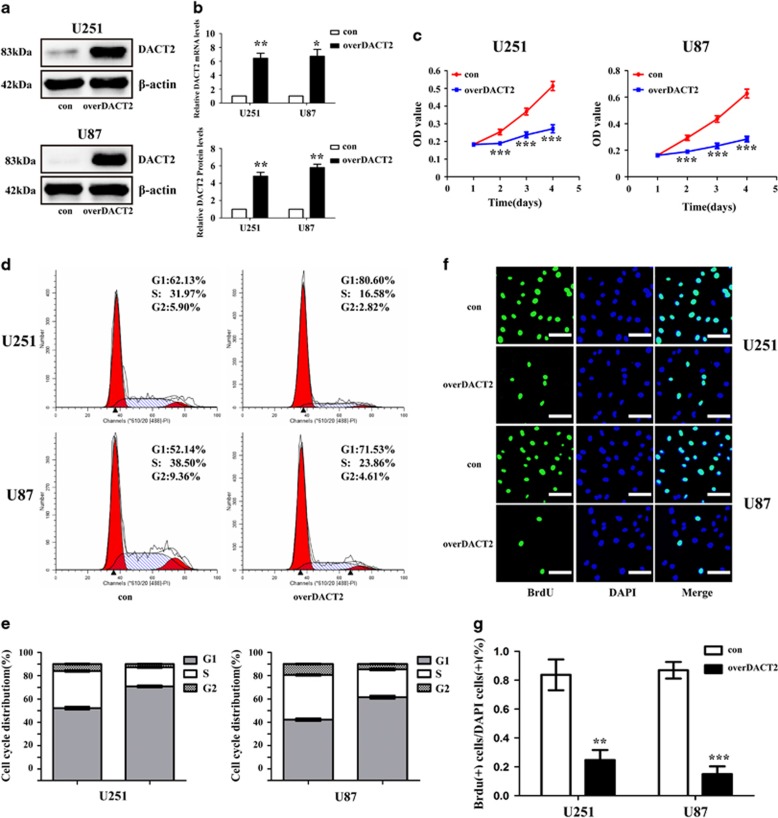
Upregulation of DACT2 inhibits proliferation of glioma cells. (**a** and **b**) qRT- PCR and western blot were conducted to determine the mRNA levels and protein of DACT2 in U251 and U87 cells. (**c**) Cell proliferation was detected in U251 and U87 cells by CCK-8 assay. (**d** and **e**) Cell cycle distributions were tested in U251 and U87 cells by Flow cytometry. (**f** and **g**) Glioma cells were stained with BrdU (green) and DAPI (blue), scale bars: 100 *μ*m (200 ×). con: transfected with empty vectors; overDACT2: transfected with DACT2 vectors. (*P<0.05, **P<0.01, ***P<0.001)

To further examine the biological effect of DACT2 on glioma cell, we overexpressed DACT2 in the glioma cell lines. qRT-PCR and western blot analysis were used to evaluate the expression levels of DACT2 in four glioma cell lines (U87, U251, SHG44 and A172; Figures 1g and h). Two glioma cells (U251 and U87) that showed low expression of DACT2 were selected for overexpressing experiments via a lentivirus-based method and SHG44 and A172, which showed high expression of DACT2 were used for knockdown experiments. The expression of DACT2 was confirmed by qRT-PCR and western blot analysis (Figures 2a and b and Supplementary Figure 1A and B). We next investigated the cell proliferation of these glioma cells by CCK8 and colony formation assays. The CCK8 results showed a striking reduce in the proliferation and indicated that cell proliferation was inhibited *in vitro* after DACT2 overexpression (Figure 2c). On the other hand, knockdown of DACT2 significantly increased the A172 and SHG44 cells proliferation (Supplementary Figure 1C). In addition, BrdU immunofluorescence analysis was also used to investigate the proliferation of glioma cells. In line with the results of CCK8, BrdU positive cells decreased in DACT2 overexpressing glioma cells (Figures 2f and g). Therefore, these findings indicated that DACT2 inhibits proliferation in glioma cells *in vitro*.

### Overexpression of DACT2 induces cell cycle arrest and apoptosis of glioma cells

The effect of DACT2 overexpressing on cell cycle was assessed by the Flow cytometry assay. The results showed that overexpression of DACT2 significantly increased the population of G0/G1 phase cells in DACT2 overexpressing glioma cells, and decreased S phase cells compared with control cells (Figures 2d and e). In contrast, knockdown of DACT2 decreased the percentages of G1 phase cells and increased the percentages of S phase (Supplementary Figure 1D and E).

**Figure 3:**
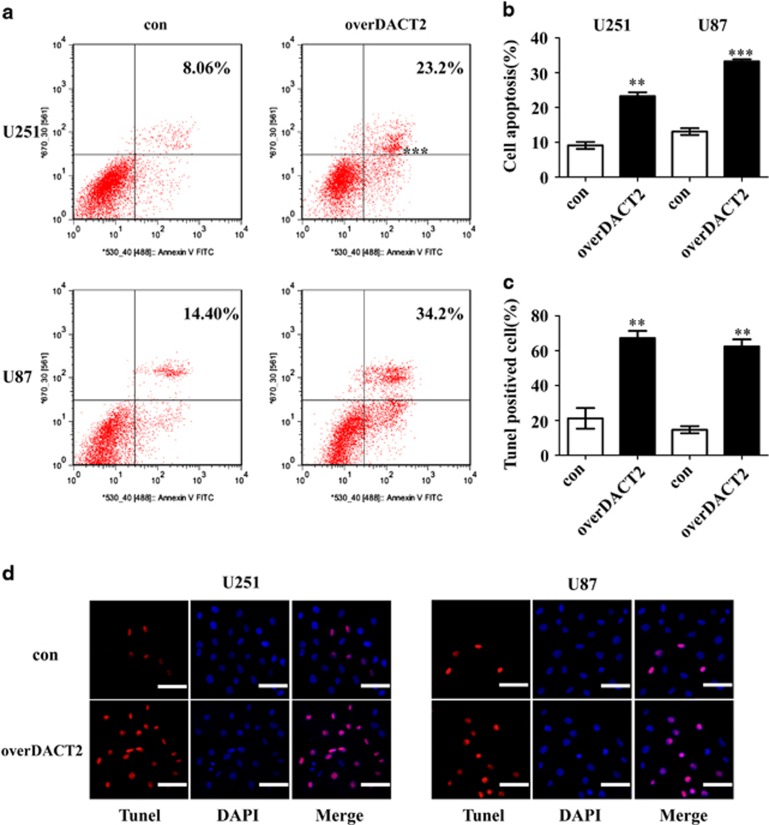
Overexpression of DACT2 promotes apoptosis of glioma cells. (**a** and **b**) Flow cytometric analysis was conducted to determine cellular apoptosis using annexin V/PI double staining. (**c** and **d**) Tunel assay of con cells and overDACT2 cells observed by microscopy, scale bars: 100 *μ*m (200 ×). con: transfected with empty vectors; overDACT2: transfected with DACT2 vectors. (**P<0.01, ***P<0.001)

We next used Annexin V-FITC/PI staining and Tunel assay to evaluate the apoptotic role of DACT2 in glioma cells. Flow cytometry analysis revealed that overexpression of DACT2 in both glioma cells markedly induced apoptosis compared with control cells (Figures 3a and b) and increased tunel positive cells (Figures 3c and d). Whereas knockdown of DACT2 reduced the rate of apoptosis in glioma cell (Supplementary Figure 1F and G). These results suggested that DACT2 has a pivotal role in not only the proliferation but also in the apoptosis of glioma cells.

We also investigated glioma cells sensitivity to temozolomide (TMZ) by CCK8. The result showed that overexpression of DACT2 in U251 and U87 cells markedly reduced chemosensitivity to TMZ (Supplementary Figure 2A and B). On the other hand, DACT2 knockdown in A172 and SHG44 cells significantly increased chemosensitivity to TMZ (Supplementary Figure 2C and D). These data indicated that low expression of DACT2 rendered glioma cells to be more resistant to TMZ treatment.

### Repression of YAP signaling is potentially involved in the anticarcinogenic function of DACT2

**Figure 4:**
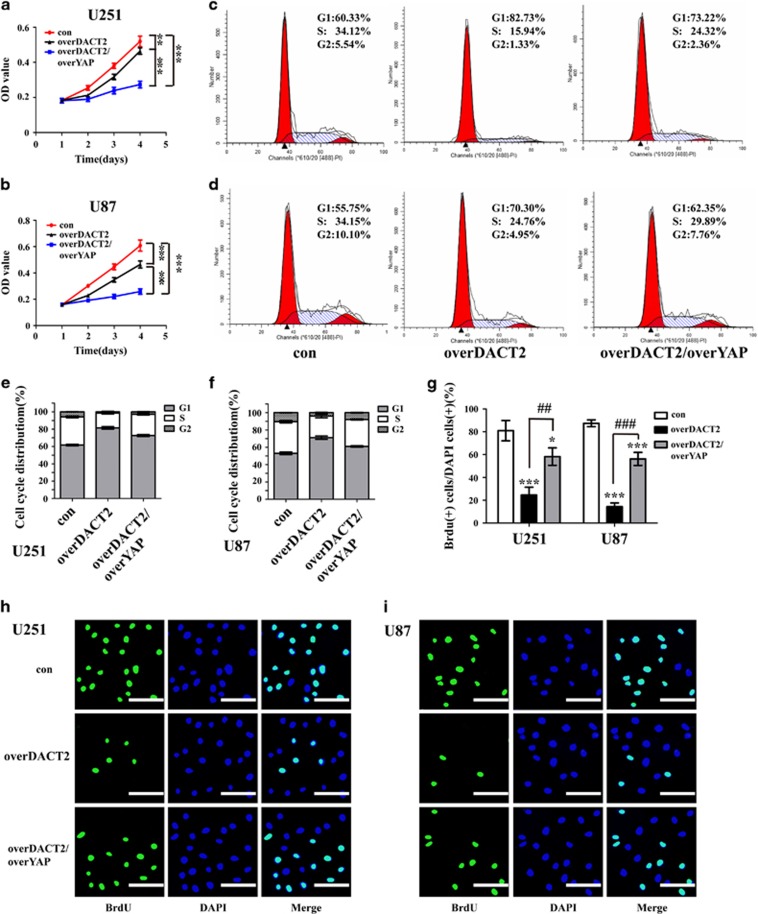
YAP rescues the proliferation-suppressive effect of DACT2 overexpressing on glioma cells. (**a** and **b**) Cell proliferation was detected in U251 and U87 cells by CCK-8 assay. (**c**–**f**) Cell cycle distributions were tested in U251 and U87 cells by Flow cytometry. (**g** and **h**) Glioma cells were stained with BrdU (green) and DAPI (blue), scale bars: 100 *μ*m (200 ×). con: transfected with empty vectors; overDACT2: transfected with DACT2 vectors, overDACT2/overYAP: transfected with DACT2 vectors and YAP vectors (*P<0.05, **P<0.01, ***P<0.001, ^##^*P*<0.01, ^###^*P*<0.001)

**Figure 5:**
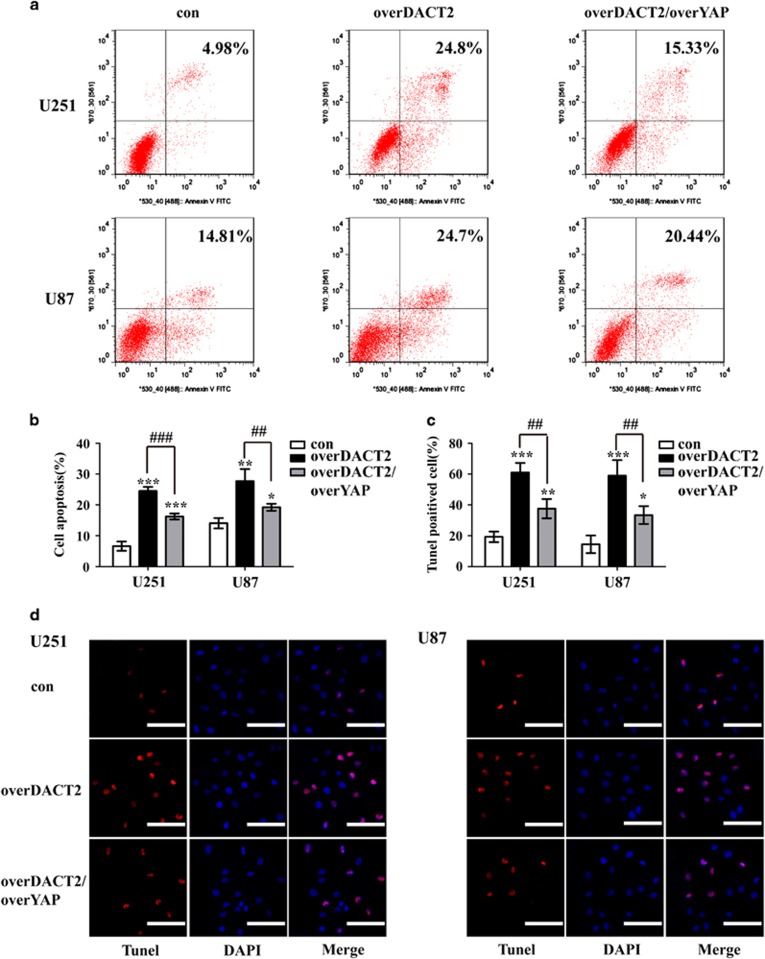
YAP rescues the apoptosis-induced effect of DACT2 overexpressing on glioma cells. (**a** and **b**) Flow cytometric analysis was conducted to determine cellular apoptosis using annexin V/PI double staining. (**c** and **d**) Tunel assay of con cells, overDACT2 cells and overDACT2/overYAP cells observed by microscopy, scale bars: 100 *μ*m (200 ×). con: transfected with empty vectors; overDACT2: transfected with DACT2 vectors, overDACT2/overYAP: transfected with DACT2 vectors and YAP vectors. (*P<0.05, **P<0.01, ***P<0.001, ^##^*P*<0.01, ^###^*P*<0.001)

**Figure 6:**
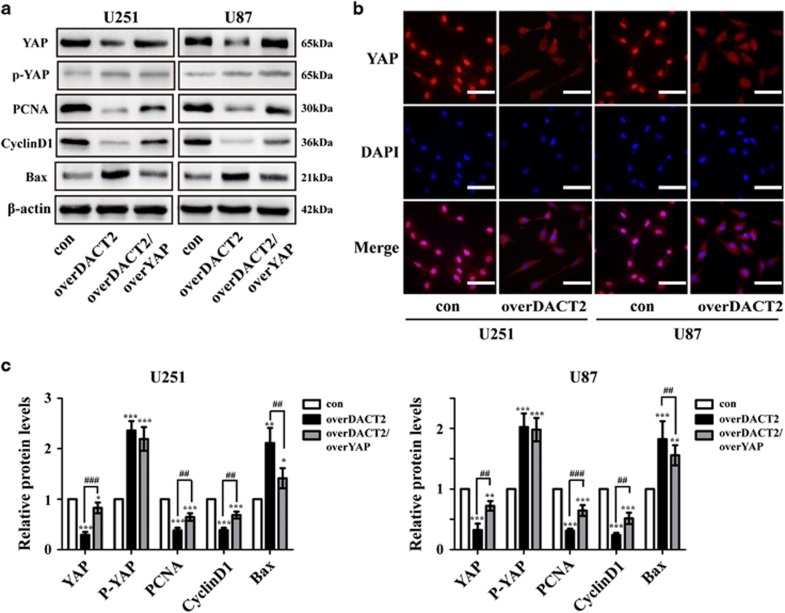
Mechanisms of DACT2 exert their functions in glioma cells. (**a** and **c**) Western blotting analysis of the YAP, p-YAP, PCNA, CyclinD1 and Bax expression in YAP-transfected U251 and U87 cells treated with DACT2. (**b**) Immunofluorescence analysis of the location of YAP in U251 and U87 cells transfected with DACT2. con: transfected with empty vectors; overDACT2: transfected with DACT2 vectors, overDACT2/overYAP: transfected with DACT2 vectors and YAP vectors (*P<0.05, **P<0.01, ***P<0.001, ^##^*P*<0.01, ^###^*P*<0.001)

To determine whether YAP is involved in DACT2’s regulation of glioma cells proliferation and apoptosis, we performed rescue experiments. Glioma cells were cotransfected with overDACT2 and overYAP, and the ability of overDACT2 was partially reversed. Cell proliferative ability was assessed by CCK8 (Figures 4a and b), and BrdU immunofluorescence analysis (Figures 4g and h). Cell cycle distribution was evaluated by Flow cytometry assay (Figures 4c–f). Annexin V-FITC/PI staining (Figures 5a–c) and Tunel assay (Figures 5d and e) were used to assess cell apoptosis. CCK8 was used for assessing glioma cells sensitivity to temozolomide (Supplementary Figure 2E and F). And YAP downstream factors related to cell proliferation, cell cycle and apoptosis were detected by Western blot (Figures 6a and c). Immunofluorescence analysis was performed to determine the location of YAP in U251 and U87 cells transfected with DACT2 (Figure 6b). All these results revealed that DACT2 not only reduced the expression of YAP, PCNA and CyclinD1 and increased Bax and p-YAP expression, but also inhibited nuclear transfer of YAP. Meanwhile, YAP overexpression could partially reverse the DACT2-induced biological effect, including cell growth inhibition, apoptosis induction, improvement of chemosensitivity and attenuated the inhibitory effects of DACT2 induction on the expression of YAP, PCNA, CyclinD1 and enhanced effects of DACT2 induction on the expression of Bax and p-YAP.

### DACT2 suppresses YAP though Wnt/*β*-catenin signaling pathway but not Hippo signaling pathway

**Figure 7:**
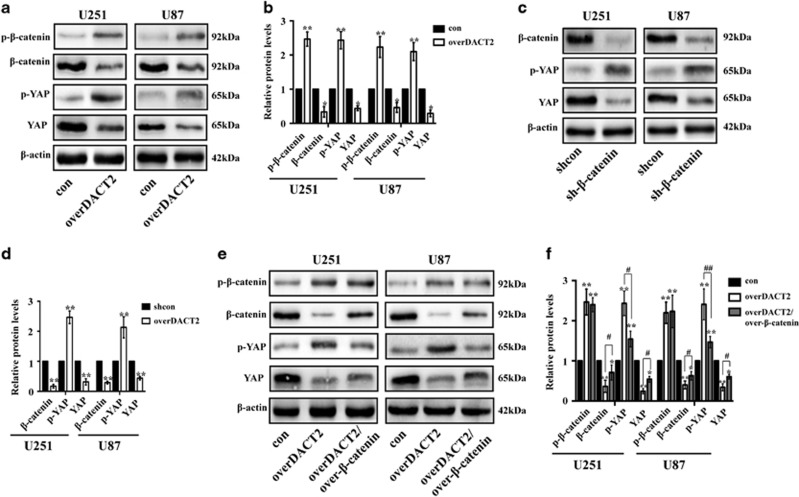
DACT2 suppresses YAP signaling pathway though Wnt/*β*- catenin signaling pathway. (**a** and **b**) Western blotting analysis of the p-*β*-catenin, *β*-catenin, p-YAP and YAP expression in overDACT2 U251 and U87 cells. (**c** and **d**) Western blotting analysis of the p-YAP and YAP expression in sh-*β*-catenin U251 and U87 cells. (**e** and **f**) Western blotting analysis of the p-*β*-catenin, *β*-catenin, p-YAP and YAP expression in *β*-catenin-transfected U251 and U87 cells treated with DACT2. con: transfected with empty vectors; overDACT2: transfected with DACT2 vectors, overDACT2/over-*β*-catenin: transfected with DACT2 vectors and *β*-catenin vectors, shcon: transfected with control vectors, sh-*β*-catenin: transfected with sh-*β*-catenin vectors (*P<0.05, **P<0.01, ^#^*P*<0.05, ^##^*P*<0.01)

**Figure 8:**
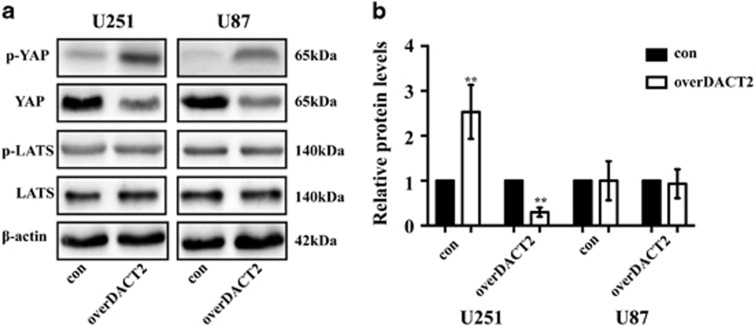
The effect of DACT2 on the Hippo signaling pathway. (**a** and **b**) Western blotting analysis of the p-YAP, YAP, p-LATS1 and LATS expression in overDACT2 U251 and U87 cells. con: transfected with empty vectors; overDACT2: transfected with DACT2 vectors (**P<0.01)

Previous studies showed DACT2 can suppress the Wnt/*β*-catenin signaling pathway that plays an important function in development of various tumors.^21, 22^ Therefore, we test the expression of p-YAP and p-*β*-catenin in overDACT U87 and U251 by Western blot (Figures 7a and b). Indeed, the expression of p-*β*-catenin and p-YAP was increased, however, the expression of *β*-catenin and YAP was reduced. Subsequently, we investigated whether *β*-catenin is required for DACT2-mediated YAP silencing. The expression of YAP was downregulated and p-YAP was increased in the sh-*β*-catenin U87 and U251 glioma cells compared with the scramble groups (Figures 7c and d). Furthermore, the results showed that the forced expression of *β*-catenin reversed DACT2-induced decrease of YAP and increase of p-YAP (Figures 7e and f). It was reported that the expression of YAP can be regulated by Hippo signaling pathway. LATS1 which is an important signaling molecule in Hippo pathway can phosphorylates YAP on serine residues which cause inactivation of YAP.^44^ Therefore, we investigate whether DACT2 suppress the expression of LAST1. The results showed that the expression of LATS1 and p-LATS1 is no difference between overDACT2 glioma cell groups and control groups (Figures 8a and b). These results above demonstrate that DACT2 suppresses YAP signaling pathway though suppressing Wnt/*β*-catenin signaling pathway but not Hippo signaling pathway.

### DACT2 suppresses the growth of the glioma *in vivo*

**Figure 9:**
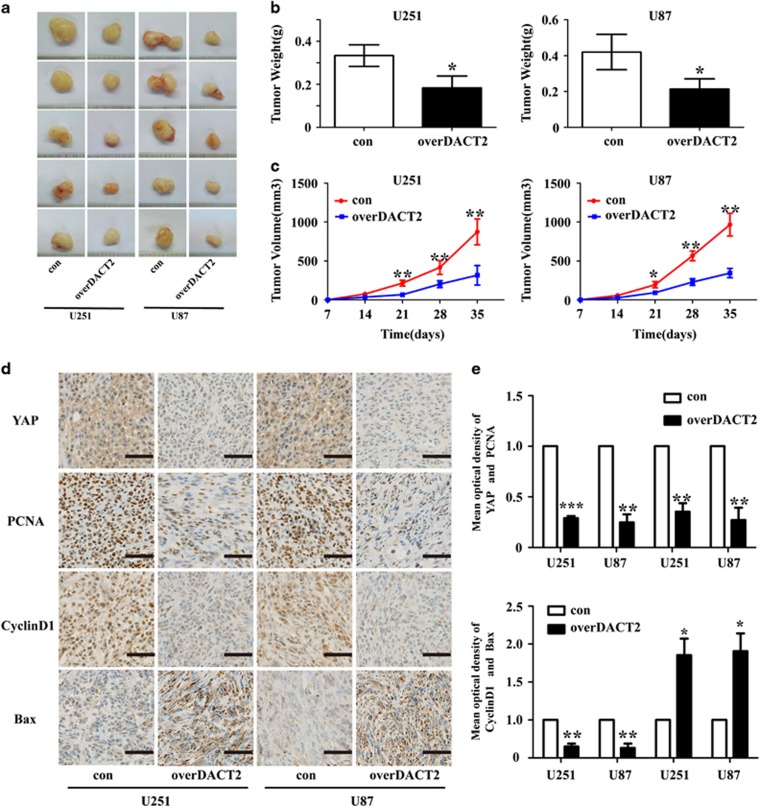
The growth-suppressive effect of DACT2 overexpressing on glioma cells *in vivo*. (**a**) DACT2 inhibited the growth of U251 and U87 cells *in vivo*. (**b** and **c**) Tumor weight and growth curve. (**d** and **e**) IHC analysis of the protein expression of YAP, PCNA, CyclinD1 and Bax in transplanted tumors, scale bars: 50 *μ*m (400 ×). con: transfected with empty vectors; overDACT2: transfected with DACT2 vectors (*P<0.05, **P<0.01, ***P<0.001)

Furthermore, we investigated the effects of DACT2 on tumor growth *in vivo*. The nude mice were inoculated with glioma cells. Compared with the control group, tumor growth and weight in the DACT2 overexpressing group were markedly suppressed (Figures 9a–c). In contrast, DACT2 knockdown promoted growth and enhance the weight of glioma (Supplementary Figure 3A–C). In addition, IHC assay revealed that DACT2 overexpression upregulated the expression of Bax and downexpressed the expression of YAP, PCNA and CyclinD1 and in glioma tissues (Figures 9d and e), and DACT2 knockdown caused opposide result (Supplementary Figure 3D and E). Collectively, these data indicated that DACT2 has a negative effect on the growth of human glioma cells *in vivo*.

## Discussion

In the current study, we found that DACT2 was frequently downexpressed in the glioma tissues in comparison to normal brain tissues by qRT-PCR, western blot and immunohistochemical analysis. Moreover, expression level of the DACT2 inversely associated with tumor grade. This was in agreement with previous findings that DACT2 in many other tumor tissues was downexpressed in comparison to the matched normal tissues because of DACT2 promoter methylation.^16, 17, 18, 19, 20, 21, 22^ This finding further supported the concept that DACT2 represented a novel tumor suppressor gene. We further hypothesized that the DACT2 may suppress progression of glioma. To test this hypothesis, we analyzed the expression of DACT2 and the clinicopathologic features through TCGA glioma data set, as well as the biological significance of DACT2 in glioma cell lines. Data of TCGA also showed that expression of DACT2 was lower in higher grade glioma. Reduced DACT2 expression markedly affected the outcome of patients with glioma, and was correlated with poor survival. Clinicopathological analysis implied that downexpression of DACT2 was associated with the WHO grade, KPS scale and age. Furthermore, Univariate and multivariate analyses indicated that DACT2 was an independent prognostic factor for the OS of patients with glioma.

Limitless proliferation and resistance to apoptosis are the remarkable hallmarks of malignancies. Abnormalities in cell apoptosis and cell cycle also result in deregulation of tumor cell proliferation.^6, 45, 46, 47^ According to our data, DACT2 significantly inhibited proliferation, enhanced chemosensitivity to TMZ and induced G1/S arrest, apoptosis of glioma cells. In addition, xenograft model also revealed that the DACT2 markedly reduced the growth of glioma. These results were consistent with the roles of DACT2 on other tumor cells.^19, 20, 21^ Thus, the decreased expression of DACT2 may be in relation to the unstrained proliferation and resistance to apoptosis potential of glioma cells.

The accurate mechanism that DACT2 regulates proliferation and apoptosis of glioma cells remains unclear. YAP as a key downstream transcription factor of the Hippo signaling pathway plays a critical role in cell proliferation and apoptosis.^23, 24^ In addition, overexpression of YAP correlated with poor overall survival of glioma patients.^36^ However, little is known about whether YAP is involved in DACT2’s regulation of glioma cells proliferation and apoptosis. In the present study, we found that YAP overexpression could partially reverse the DACT2-induced biological effect, including cell growth inhibition and apoptosis induction. Furthermore, we found that overexpression of DACT2 inhibited cell proliferation and apoptosis via downregulation of YAP, PCNA and CyclinD1 expression and upregulation of p-YAP expression and Bax. Notably, overexpression of DACT2 prevented YAP translocating into nucleus and sequestering in the cytoplasm to degrade.

YAP is the key downstream transcription factor of the Hippo signaling and can be regulated by LATS1 which play an important role in Hippo signaling pathway.^44^ It was reported that DACT2 could suppress Wnt/*β*-catenin signaling pathway and inhibit p-*β*-catenin expression.^22^ Therefore, we investigated whether DACT2 suppress YAP though Wnt/*β*-catenin signaling pathway or Hippo signaling pathway or both them. Our results demonstrate DACT2 can suppresses YAP through Wnt/*β*-catenin signaling pathway but not Hippo signaling pathway.

In conclusion, we showed for the first time that DACT2 was downexpressed in gliomas and decreased DACT2 was correlated with glioma grade and poor survival. Meanwhile, the present study provides evidence that DACT2 as a tumor suppressor gene could inhibit growth and induce apoptosis of glioma cells by suppressing YAP through Wnt/*β*-catenin signaling pathway. Our findings suggest that DACT2 is a novel tumor suppressor gene and may be a potential therapeutic target in gliomas.

### Ethics statement

All procedures performed in studies involving human participants were in accordance with the ethical standards of the local, independent ethics committee at The Second Affiliated Hospital of Chongqing Medical University Hospital. Written informed consent was obtained from all patients. All procedures performed in studies involving animals were in accordance with the ethical standards of the local, independent ethics committee at Chongqing Medical University. Informed consent was obtained from all individual participants included in the study.

## Materials and methods

### Patients and tissue preparation

A total of 80 glioma and 10 non-neoplastic brain samples were obtained from between 2008 and 2012 in the Second Affiliated Hospitals of Chongqing Medical University. In addition, eight glioma tissues and the corresponding adjacent non-neoplastic tissues were collected in 2016. None of the patients had received prior chemotherapy or radiotherapy. The patients’ clinical characteristics such as age, gender, and WHO grade, were collected. Patients’ consent and approval from the Institutional Research Ethics Committee of Chongqing Medical University were obtained for research purposes. For histological analysis, resected glioma and non-neoplastic brain tissues were fixed in formalin, embedded in paraffin and cut into 5-*μ*m thick sections. For qRT-PCR and western blot analysis, tissues were immediately frozen in liquid nitrogen and kept at −80 °C until analysis.

### Cell culture and reagents

SHG44, U87, U251, and A172 glioma cell lines were from Shanghai Life Academy of Sciences Cell Library. The four glioma cell lines were maintained in a 5% CO2 atmosphere at 37 °C in DMEM supplemented with 100U/ml penicillin, 100 μg/ml streptomycin (Hyclone) and 10% FBS. Antibodies against DACT2 were obtained from OriGene, phospho-YAP, YAP, p-*β*-catenin, *β*-catenin, p-LATS1, LATS1, PCNA, Bax, and CyclinD1 were obtained from Cell Signaling Techology (Danvers, MA, USA). *β*-actin was purchased from KangCheng Biotech (Shanghai, China).

### Immunohistochemistry

Tissue sections were cut and mounted on slides. After de-waxing and rehydration, the sections were antigen-retrieved in 10 mm citrate buffer for 5 min at 100 °C. Endogenous peroxidase activity and non-specific antigens were blocked with 3% hydrogen peroxide and serum, followed by incubation with DACT2 antibody overnight at 4 °C. Slides were then incubated with goat anti-rabbit secondary antibody, developed using 3,3-diaminobenzidine (DAB) solution and counterstained with hematoxylin. PBS was used in place of the primary antibodies for the negative controls which were processed along with the samples. No apparent immunoreactivity was detected in negative controls. After staining, the slides were reviewed by two independent pathologists using a microscope (DM6000B). Immunohistochemical staining of DACT2 was calculated as both percentage of positive cells and color intensity. The percentage of the positivity was classified as ‘0’ (negative), ‘1’ (<10%), ‘2’ (10–50%), and ‘3’ (>50%). The intensity was graded as ‘0’ (absent), ‘1’ (light yellow), ‘2’ (yellowish brown), and ‘3’ (brown). The expression of DACT2 was evaluated by staining index (SI), which was calculated using the following formula: SI=proportion score × intensity score. SI of 0 was categorized as negative (−), 1–2 as low expression (1+), 3–4 as moderate expression (2+), 6 or 9 as high expression (3+).

### Lentivirus transfection

DACT2 lentivirus (overDACT2) used for DACT2 overexpression, YAP lentivirus (overYAP) used for YAP overexpression, *β*-catenin lentivirus (over-*β*-catenin) used for *β*-catenin overexpression, as well as lentiviral constructs expressing DACT2 shRNA (shDACT2), *β*-catenin shRNA (sh-*β*-catenin), and matched control lentivirus (shcon) and negative control lentivirus (con) were purchased from Genechem Co., Ltd (Shanghai, China). Glioma cells stably expressing the DACT2 shRNA targeting the sequence 5′-GTGCCAAGCTGTGCCGTATT-3′ and *β*-catenin shRNA targeting the sequence 5′-ATCTGTCTGCTCTAGTAATAA-3′. One day before transfection, 5 × 10^4^cells per well (reaching about 30% confluency at the time of transfection) were cultured in six-well plates. These lentiviruses were introduced into glioma cells treated with 8 ug/ml polybrene (Genechem) and complete medium. Transfection effects were observed by a fluorescence microscope after 48 h. Puromycin or neomycin was used to purify these infected cells. Effective forced expression of DACT2 was monitored by real-time polymerase chain reaction (PCR) and western blot analysis after 72 h.

### Quantitative real-time PCR

Total RNA in cells and tissues were extracted using RNAiso Plus (TaKaRa, Beijing, China). The concentrations of these RNA samples were then measured using a spectrophotometer and the RNA specimens were reverse-transcribed into cDNA using the Primescript RT reagent Kit (TaKaRa). The primer sequence and product size for DACT2 was: forward 5′-TGATCAATGTGGACGCCGGGC-3′ and reverse 5′-GTCGACTCACACCATGGTCATGAC-3′, and for *β*-actin, which was used as a standard, was: forward 5′-TTCCAGCCTTCCTTCCTGGG-3′ and reverse 5′-TTGCGCTCAGGAGGAGCAAT-3′. Amplification conditions were as follows: 95 °C for 30 s, followed by 40 cycles at 95 °C for 15 s, and 60 °C for 45 s. The relative fold-changes in mRNA levels were calculated according to the 2^−ΔΔCT^ method.^41^

### Western blot

The tissues and cells were lysed by RIPA Lysis Buffer (Beyotime Institute of Biotechnology, Beijing, China) containing PMSF and phosphatase inhibitor. An equal amount of each protein sample was separated by 8–12% SDS–PAGE and transferred onto PVDF membranes. After incubation with primary antibodies overnight at 4 °C including DACT2(1:1000), total YAP (1:500), phospho-YAP (1:250), *β*-catenin (1:500), phospho-*β*-catenin (1:300), phospho-LATS1 (1:300), LATS1 (1:1000), *β*-actin (1:800), PCNA (1:500), CyclinD1 (1:500) and Bax (1:500), the PVDF membranes were washed three times with TBST buffer, and incubated with secondary antibody (1:5000) for 1 h at 37 °C. Then the membranes were washed three times in TBST buffer and the amount of protein in each band was quantified using the Quantity One 4.6 computer software (Bio-Rad, Hercules, CA, USA).

### Cell proliferation assay

Cells were seeded into 96-well plates at a density of 2000 cells/well and incubated at 37 °C. For 24, 48, 72 and 96 h, 10 *μ*l of Cell Counting Kit-8 (Beyotime) was added to each well and the cells were incubated for 1 h at 37 °C. The absorbance values were read at 450 nm using an enzyme-labeled instrument.

### Flow cytometric assay to detect cell cycle and apoptosis

A total of 5 × 10^5^ cells were collected and fixed in 70% icecold ethanol overnight. Cells were incubated with 10 mg/ml RNase (Sigma, St. Louis, MO, USA) and 50 mg/ml propidium iodide (Sigma) at 37 °C for 30 min in the dark. The cell cycle was analyzed by flow cytometry (BD Bioscience, San Jose, CA, USA).

To determine cell apoptosis, the same treated cells were harvested and incubated with reagents from the Annexin V-FITC apoptosis kit (BioVision, Wuhan, China) according to the manufacturer’s protocol.

### Tunel assay

Cells were plated on coverslips for 24 h. Cells were washed with PBS once, then fixed with 4% paraformaldehyde for 20 min, blocked with 5% goat serum at 37 °C for 30 min and then treated with 0.3% Triton X-100–PBS for 10 min at room temperature. An enzyme solution was added to the label solution (1:1 dilution) to obtain a terminal deoxynucleotidyl transferase-mediated dUTP-biotin nick end labeling (Tunel) reaction mixture (Beyotime), of which 50 *μ*l were added to each sample and incubated for 60 min at 37 °C in a humidified atmosphere in the dark. Microscope (DM6000B; Leica, Wetzlar, Germany) was used to automatically visualize images of the cells.

### BrdU immunofluorescence assay

Cells were grown on coverslips in the 6-well plates (2 × 10^4^cells/well) overnight. The BrdU (Sigma) stock solution at 10 mg/ml was diluted 1000 × in the culture medium and incubated for 30 min. Cells were washed with PBS, fixed in 4% paraformaldehyde for 20 min, and permeabilized with 2 M HCl for 15 min and 1% Triton X-100 for 15 min. The cells were blocked with 10% goat serum for 1 h, incubated with a primary rat antibody against BrdU (1 : 300, Abcam, Cambridge, MA, USA) for 2 h, and then incubated with the secondary antibody Alexa Fluor 488 (1 : 500, Invitrogen, Waltham, MA, USA) for 2 h. Incubation with 300 nM DAPI for 15 min was used for counterstaining. Microscope (DM6000B) was used to automatically visualize images of the cells.

### Immunofluorescence

Cells were plated on coverslips for 24 h. Cells were fixed with 4% paraformaldehyde, incubated in 0.3% Triton X-100 for 15 min, followed by blocking with 5% goat serum. The cells were then incubated with primary antibodies against YAP at 4 °C overnight, followed by appropriate secondary antibodies (Alexa Fluor 555; Bioss). The nuclei were counterstained with DAPI (Beyotime). The immunofluorescent signals were detected by Microscope (DM6000B).

### Chemosensitivity assay

Glioma cells were seeded at a density of 3000 cells per well in a 96-well plate overnight. Temozolomide (Sigma) was added with the nal concentration ranging from 25 to 400 *μ*M. Forty-eight hours later, cell viability was assayed using a CCK8 kit.

### Xenograft tumor model

The male nude mice (4 weeks old) used in this study were provided by experimental animal center of Chongqing Medical University. All animal studies were approved by the Ethics Committee of Chongqing Medical University. The glioma cells were re-suspended in DMEM at a density of 2 × 10^6^ cells per 50 *μ*l and were injected subcutaneously into the nude mice. Tumor volumes were recorded at 7, 14, 21, 28 and 35 days after inoculation according to the formula described previously.^42^ After that, the mice were killed and tumor tissues were excised and weighed. The excised tumor tissues were used for immunohistochemistry.

### The cancer genome atlas analysis

Level 3 RNA-seq data of LGG and GBM patients were downloaded from the publicly available TCGA data portal website (http://cancergenome.nih.gov). Corresponding clinical data, including survival time, survival status, gender, age, KPS and grade were also obtained from TCGA. Patients with incomplete clinical data were excluded from this analysis.

### Statistical analysis

Statistical analyses were performed using SPSS 17.0 (Chicago, IL, USA). Statistical differences among groups were analyzed by ANOVA, Kruskal–Wallis, *t*-test or *χ*2-square test. The prognostic significance analysis was performed using Kaplan–Meier method and log-rank tests. Cox’s proportional hazards model was used to identify the factors with an independent influence on survival. *P*<0.05 was considered to be statistically significant. All the data are presented as mean±SD.
