# Supplementary material for: Upregulation of DACT2 suppresses proliferation and enhances apoptosis of glioma cell via inactivation of YAP signaling pathway
Source: Cell Death Dis. 2017 Aug 10;8(8):e2981–. doi: 10.1038/cddis.2017.385 (PMC5596571; doi:10.1038/cddis.2017.385)
Supplement: Supplementary Table 1 [file cddis2017385x1.doc]

| Groups |  | Number | DACT2 expression | | *P* |
| --- | --- | --- | --- | --- | --- |
|  |  |  | Low expression | High expression |  |
| Gender |  |  |  |  |  |
| Male |  | 235 | 117 | 118 | 0.923 |
| Female |  | 193 | 97 | 96 |
| Age(yeas) |  |  |  |  |  |
| >65 |  | 59 | 41 | 18 | <0.01 |
| ≤65 |  | 369 | 173 | 196 |
| WHO grade |  |  |  |  |  |
| II |  | 141 | 55 | 86 | <0.01 |
| III |  | 164 | 72 | 92 |
| IV |  | 123 | 87 | 36 |
| KPS |  |  |  |  |  |
| >80 |  | 222 | 96 | 126 | <0.01 |
| ≤80 |  | 206 | 118 | 88 |

**Supplementary Table1 Relationship between DACT2 expression with clinicopathological features**

KPS : Karnofsky Performance Score, WHO: World Health Organization.
