# Supplementary material for: Upregulation of DACT2 suppresses proliferation and enhances apoptosis of glioma cell via inactivation of YAP signaling pathway
Source: Cell Death Dis. 2017 Aug 10;8(8):e2981–. doi: 10.1038/cddis.2017.385 (PMC5596571; doi:10.1038/cddis.2017.385)
Supplement: Supplementary Table 2 [file cddis2017385x2.doc]

| Variable | Univariate analysis, *p* value | Multivariate analysis, *p* value | Relative risk |
| --- | --- | --- | --- |
| Age  ( >65 and ≤65) | <0.01 | <0.01 | 1.04 |
| Gender  (male and Female) | <0.05 | 0.26 | 1.19 |
| WHO grade  (II, III and IV) | <0.01 | <0.01 | 3.42 |
| KPS  (>80 and ≤80 ) | <0.01 | <0.01 | 0.98 |
| DACT2 expression  (high and low) | <0.01 | <0.01 | 1.58 |

**Supplementary Table2 Univariate and multivariate analysis of prognostic parameters in patients with gliomas using Cox regression analysis**

KPS = Karnofsky Performance Score, WHO = World Health Organization.
