# Supplementary material for: Upregulation of DACT2 suppresses proliferation and enhances apoptosis of glioma cell via inactivation of YAP signaling pathway
Source: Cell Death Dis. 2017 Aug 10;8(8):e2981–. doi: 10.1038/cddis.2017.385 (PMC5596571; doi:10.1038/cddis.2017.385)
Supplement: Supplementary Figure Legends [file cddis2017385x6.docx]

**Supplementary Figure Legends**

**Supplementary Figure 1: Knockdown of DACT2 promotes proliferation and inhibits apoptosis of glioma cells**.

A., B. qRT- PCR and western blot were conducted to determine the mRNA levels and protein of DACT2 in A172 and SHG44 cells. C. Cell proliferation was detected in A172 and SHG44 by CCK-8 assay. D., E. Cell cycle distributions were tested in A172 and SHG44 by Flow cytometry. F. Flow cytometric analysis was conducted to determine cellular apoptosis using annexin V/PI double staining. shcon: transfected with control vectors, shDACT2: transfected with shDACT2 vectors (**P*<0.05, ***P* < 0.01, ****P* < 0.001).

**Supplementary Figure 2: DACT2 contributes to glioma cell temozolomide sensitivity.**

A., B. Cell proliferation was examined in U87 and U251 cells (A) A172 and SHG44 cells (B) treated with TMZ at different doses. C. Cell proliferation was examined in YAP-transfected U251 and U87 cells treated with DACT2. The CCK8 assay was conducted 48 hours after TMZ treatment. overDACT2: transfected with DACT2 vectors, overDACT2/overYAP: transfected with DACT2 vectors and YAP vectors. shcon: transfected with control vectors, shDACT2: transfected with shDACT2 vectors (**P*<0.05, ***P* < 0.01, ****P* < 0.001).

**Supplementary Figure 3:**  Knockdown of DACT2 promotes the growth of glioma cells in vivo.

A. Knockdown of DACT2 promoted the growth of A172 and SHG44 cells in vivo. B., C. Tumor weight and growth curve. D., E. IHC analysis of the protein expression of YAP, PCNA, CyclinD1 and Bax in transplanted tumors, scale bars: 50 μm (400×). shcon: transfected with control vectors, shDACT2: transfected with shDACT2 vectors. (**P*<0.05, ***P* < 0.01, ****P* < 0.001).
